# Ccdc6 knock-in mice develop thyroid hyperplasia associated to an enhanced CREB1 activity

**DOI:** 10.18632/oncotarget.3858

**Published:** 2015-04-27

**Authors:** Vincenza Leone, Concetta Langella, Francesco Esposito, Claudio Arra, Giuseppe Palma, Domenica Rea, Orlando Paciello, Francesco Merolla, Davide De Biase, Serenella Papparella, Angela Celetti, Alfredo Fusco

**Affiliations:** ^1^ Istituto per l' Endocrinologia ed Oncologia Sperimentale del CNR e/o Dipartimento di Medicina Molecolare e Biotecnologie Mediche, Università degli Studi di Napoli “Federico II”, Naples, Italy; ^2^ Istituto Nazionale per lo Studio e la Cura dei Tumori “Fondazione Giovanni Pascale”, IRCCS, Naples, Italy; ^3^ Department of Pathology and Animal Health, University of Naples “Federico II”, Naples, Italy; ^4^ Instituto Nacional de Câncer - INCA, Rua André Cavalcanti, Rio de Janeiro, CEP RJ, Brazil

**Keywords:** Ccdc6, thyroid, CREB1, knock-in mice

## Abstract

CCDC6 was originally identified upon rearrangement with RET in human thyroid papillary carcinomas generating the RET/PTC1 oncogene. We have previously reported that CCDC6 interacts with CREB1 and represses its transcriptional activity.

Since the function of at least one allele of CCDC6 is lost following RET/PTC1 rearrangements, we aimed at the generation of mice, carrying a CCDC6 mutant gene. Previous studies suggested that the coiled-coil domain of CCDC6, mainly encoded by human exon 2, is required for the protein function. Therefore, we engineered a murine Ccdc6 construct, carrying a deletion of the exon 2, that was able to exert only a mild repression on CREB1 transcriptional activity, with respect to the wild type Ccdc6. Subsequently, we generated Ccdc6^−ex2^ knock-in mice. These mice developed thyroid hyperplasia associated with an enhanced CREB1 activity and an increased expression of the CREB-1 regulated genes.

These results strongly support a CCDC6 promoting role, ascribed to its functional impairment, in the development of thyroid papillary carcinomas harboring the RET/PTC1 oncogene

## INTRODUCTION

CCDC6 was identified upon rearrangement with RET in papillary thyroid carcinomas (PTC) generating the RET/PTC1 oncogene detectable in about 20% of PTCs [1]. This rearrangement has been recently identified also in lung adenocarcinomas even though at a much lower frequency (less than 5%) [2]. Moreover, CCDC6 gene rearrangements with genes other than RET have been reported in solid and not solid tumours [3–5] suggesting that CCDC6 gene has a high susceptibility for recombination. CCDC6 gene product is an ubiquitously expressed 65 kDa nuclear and cytosolic protein, phosphorylated by extracellular signal-regulated protein kinase following serum stimulation with no significant homology to known genes [6, 7]. Recent studies reported the involvement of this gene in apoptosis and the ability of its truncated mutant CCDC6 (1–101), which corresponds to the portion of CCDC6 included in RET/PTC1, to act as dominant negative on the wild type (wt) CCDC6-induced apoptosis [7]. Moreover, it has been shown that the CCDC6 gene product is a substrate of ATM and helps to protect genome integrity by negatively modulating PP4c activity directed towards pS139_H2AX dephosphorylation following DNA damage [8]. Recently, we demonstrated that CCDC6 is responsible for the transcriptional repression of CREB1 target genes, activating the PP1 phosphatase that is able to dephosphorylate CREB1 at Ser133. Consistently, the loss of CCDC6 keeps high levels of phosphorylated CREB in PTCs positive for the RET/PTC1 rearrangement [9].

These results support the hypothesis that even the impairment of one CCDC6 allele, following RET/PTC1 rearrangement, could contribute to papillary thyroid carcinogenesis enhancing thyroid cell proliferation through the activation of the CREB1 transcriptional activity.

In order to confirm our hypothesis we planned to generate mice carrying a mutant Ccdc6 gene. It has been demonstrated that the fragment of 60 amino acid of the CCDC6 coiled-coil domain included in the RET/PTC1 product is necessary for homo-dimerization, constitutive activation and transforming ability of the oncoprotein. Then, we hypothesized that the CCDC6 coiled-coil domain, mainly encoded by exon 2, is required for the function of the protein. Therefore, we first investigated whether the deletion of the murine exon 2 in Ccdc6 (Ccdc6-ex2) could affect the Ccdc6 ability to act as a negative modulator of the CREB1 transcriptional activity, with respect to the Ccdc6 wild type [9]. Subsequently, we proceeded to the generation of mice carrying a Ccdc6 gene deleted of exon 2 (Ccdc6-ex2 mice).

These mice developed thyroid hyperplasia likely due to an increased CREB1 activity and consequent expression of the CREB1-regulated genes, as expected from the described effect of wt CCDC6 to repress CREB1 transcriptional activity.

## RESULTS

### Ccdc6-ex2 protein increases CREB binding to CRE element but exerts a milder repression on CREB1 transcriptional activity in comparison to Ccdc6 wt

We have previously demonstrated that the CCDC6 protein was able to interact with CREB1 and modulate its activity by reducing the binding of CREB to CRE element in a dose-dependent manner [9].

**Figure 1:**
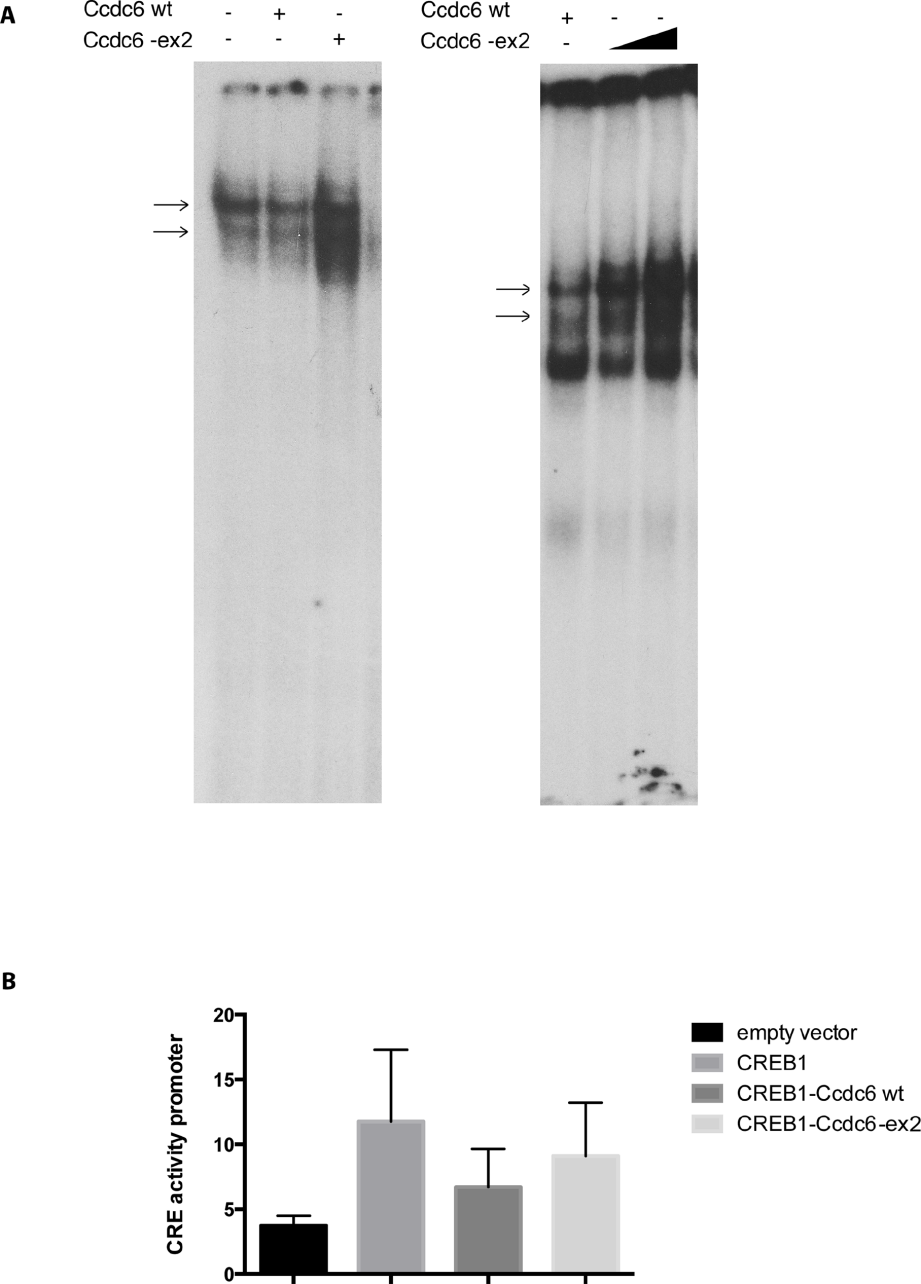
Ccdc6-ex2 protein increases CREB binding to CRE element and is less able to reduce CREB1 transcriptional activity **A.** EMSA assay was performed with a radiolabelled oligonucleotide containing the CRE element incubated with nuclear extracts from Hek-293 cells transfected with Ccdc6 wt or Ccdc6-ex2 vector (left panel) or with increasing amount of nuclear extracts (2.5 and 5 micrograms) from Hek-293 cells transfected with the Ccdc6-ex2 vector (right panel). Arrows indicate specific DNA/protein complexes. **B.** Luciferase assay was performed using the Ccdc6 wt or Ccdc6-ex2 expression constructs on the CRE-luciferase-reporter vector in Hek-293 cells. All transfections were performed in duplicate; data are mean ± SD of three independent experiments, *p* < 0.01.

Therefore, we investigated the ability of a Ccdc6 deprived of the exon 2 to repress the CREB1 transcriptional activity. We analyzed the effect of Ccdc6-ex2 on the binding of CREB to the CRE element by Electrophoretic Mobility Shift Assay (EMSA). Nuclear extracts from control cells or HeK-293 cells, overexpressing Ccdc6 wt or Ccdc6-ex2, were incubated with a CRE radiolabelled oligonucleotide. As shown in Figure 1A, nuclear proteins from Ccdc6-ex2 extracts were able to bind the CRE oligonucleotide in EMSA (left panel, lane 1). The Ccdc6-ex2 expression increased the binding of CREB to CRE element (left panel, lane 3) in a dose-dependent manner (lanes 2 and 3, right panel), compared to the expression of the Ccdc6 wt that showed a reduced binding of CREB to CRE element (left panel, lane 2). As Ccdc6-ex2 was able to increase the binding of CREB to CRE element, we analyzed the effect of Ccdc6-ex2 on CREB1 transcriptional activity. HeK-293 cells were transfected with a CRE-luc reporter gene [10] in which tandem three CRE universal sites were fused upstream to a luciferase cDNA together with Ccdc6 wt or Ccdc6-ex2 in the presence of CREB1. As shown in Figure 1B, the CREB1 overexpression enhanced the reporter CRE-luc activity more than 3-fold, whereas co-transfection of Ccdc6 resulted in a decrease of CREB1-mediated transcriptional activity. However, the inhibition of CREB1 transcriptional activity after co-transfection of the Ccdc6 with its ex2-deleted mutant construct is significantly less evident in comparison to that obtained co-transfecting the wt Ccdc6 construct. This result suggests that the deletion of the coiled-coil domain, mainly coded for by the murine exon 2, could impair the Ccdc6 protein function at least for its ability to downregulate CREB1 activity.

### Generation of *Ccdc6*-ex2 mice

**Figure 2:**
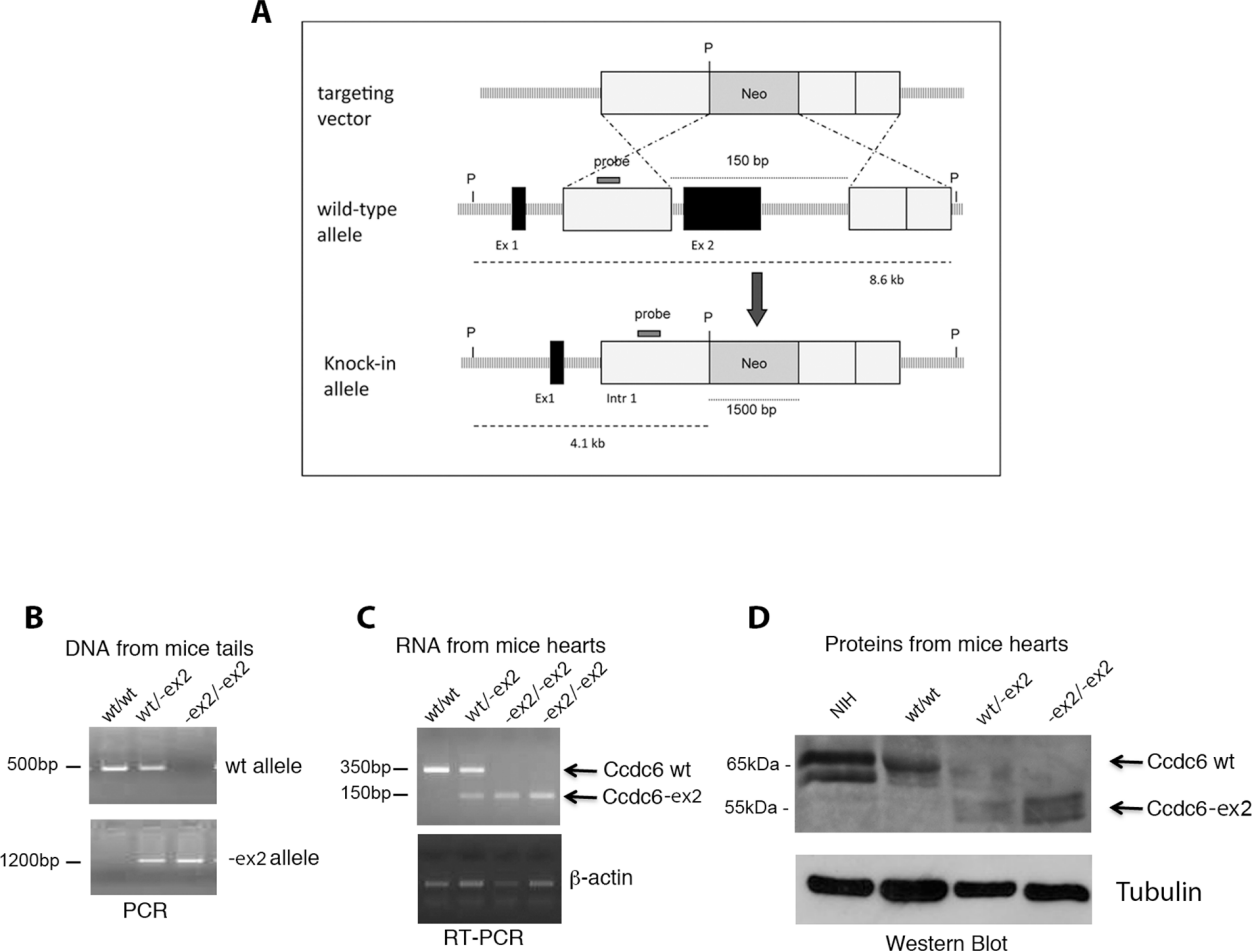
Generation of Ccdc6^−ex2/−ex2^ knock-in mice **A.** Schematic representation of the endogenous wt allele, the targeting vector and the resulting Ccdc6-ex2 allele. P, Pst1; Neo, neomycin; Ex, exon, Intr, intron. **B.** The genotyping of *Ccdc6* mice was performed using PCR analysis of DNA extracted from mice tails. **C.** RT-PCR analysis of total RNA extracted from hearts of Ccdc6 mice. **D.** Western blot analysis of Ccdc6 proteins in hearts of wt and knock-in mice. Gamma-tubulin was analyzed to evaluate equal protein loading. NIH lysate extracts have been utilized as a control of the murine Ccdc6 weight, recognized by a specific anti-CCDC6 antibody, that we generated (anti-C16-R).

These findings led us to proceed to the generation of the Ccdc6-ex2 knock-in mice. We used gene targeting techniques in embryonic stem (ES) cells to generate a mutation at the murine Ccdc6 genomic locus. We deleted exon 2 which contains most of the coiled-coil domain and replaced it with a neomycin-resistance cassette (Figure 2A) (see Materials and Methods). Cell clones resistant to G418 have been isolated and screened for Ccdc6 homologous recombination. The positive clones were expanded and injected into blastocysts from the C57BL/6 mice strains. The resulting chimaeric blastocysts were transferred to uteri of foster mothers of the same strain. The chimaeric offspring was crossed with wt mice to obtain germ line transmission with the generation of mice heterozygous for Ccdc6 gene deletion. The heterozygous mice were then crossed each other to generate homozygous mice.

PCR analysis of tail DNA (Figure 2B) confirmed the presence of an ex-2 deleted allele in Ccdc6^−ex2/−ex2^ mice. Conversely, Ccdc6^wt/wt^ mice showed only the wt allele, whereas Ccdc6^wt/−ex2^ showed both alleles. Reverse transcriptase-polymerase chain reaction (RT-PCR) of RNA obtained by Ccdc6^−ex2/−ex2^ mice hearts (Figure 2C) confirmed the expression of transcripts of the Ccdc6-ex2 alleles, whereas Ccdc6^wt/−ex2^ heterozygous mutants expressed an intermediate amount of Ccdc6-ex2 mRNA and Ccdc6^wt/wt^ did express only the wt transcript. Finally, antibodies able to recognize the COOH-region of the Ccdc6 protein revealed the expression product of the truncated product (55 kDa) in Ccdc6^−ex2/−ex2^, the expression of wt product (65 kDa) in Ccdc6^wt/wt^ mice and both the products in Ccdc6^wt/−ex2^ heterozygous mice, at immunoblot (Figure 2D). The products we observed at immunoblot appeared of a bigger size than expected, on account of post-translational modifications, that apparently still occur in ccdc6 murine products as reported for human CCDC6 protein [7, 11]. It is worth to note that heterozygote matings yielded wt, heterozygous, and homozygous offspring at not expected Mendelian ratio, at the expense of Ccdc6^−ex2/−ex2^ mice suggesting significant embryonic lethality for the Ccdc6^−ex2/−ex2^ mice (Table 1).

**Table 1:** The table indicated the frequency of wt, heterozygous, and homozygous offspring after heterozygote matings in Ccdc6 knock-in mice

	heterozygote matings offspring = 198 mice	
	N° mice	%
**Ccdc6 wt/wt**	81	41
**Ccdc6 wt/−ex2**	95	48
**Ccdc6 −ex2/−ex2**	22	11

### Ccdc6^−ex2/−ex2^ mouse embryonic fibroblasts (MEFs) showed a reduced rate of growth and an increased susceptibility to apoptosis compared to the Ccdc6 wt counterparts

**Figure 3:**
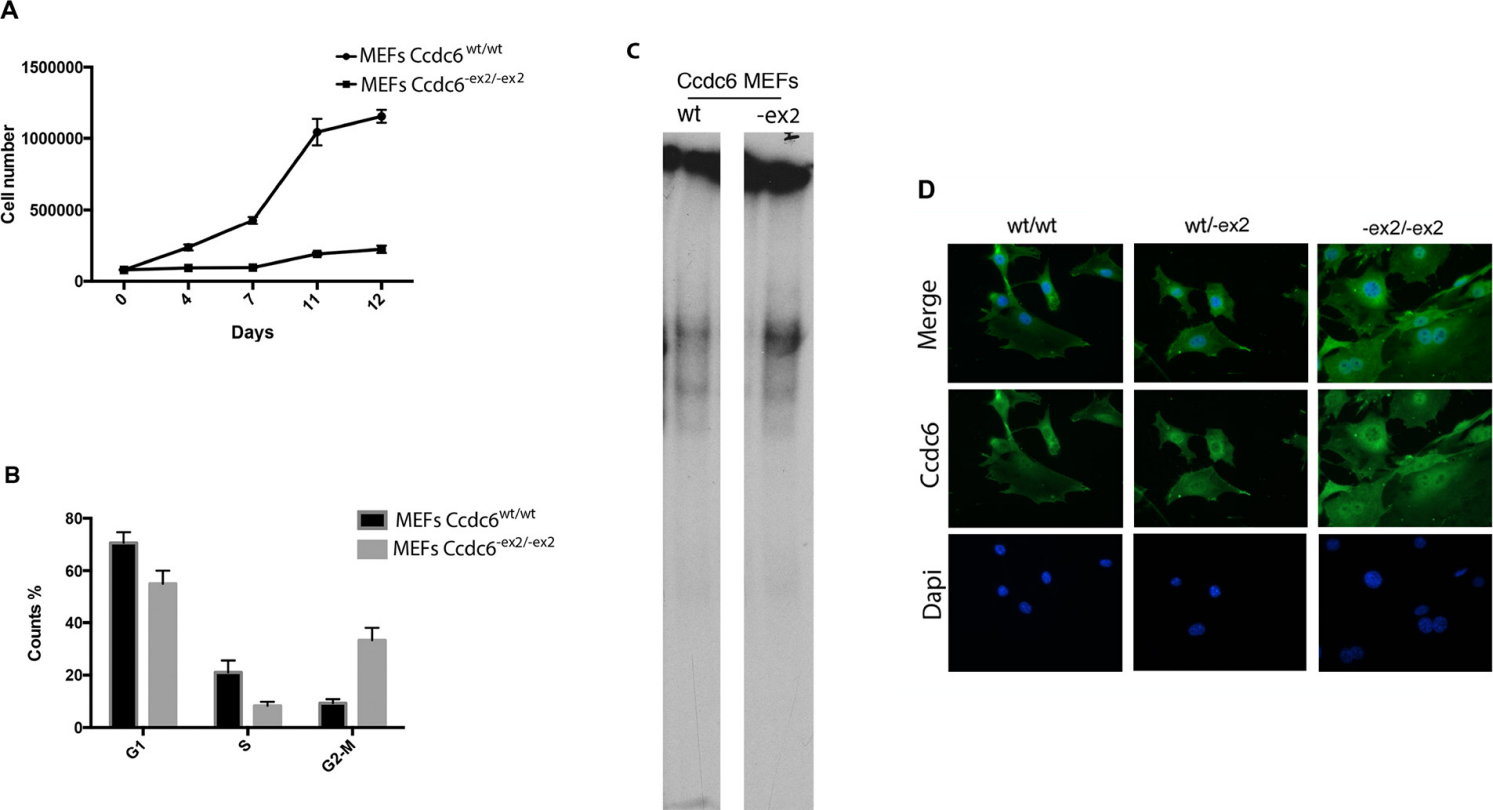
Analysis of Ccdc6^−ex2/−ex2^ and wt MEF growth **A.** MEFs were prepared from Ccdc6^wt/wt^ and Ccdc6^−ex2/−ex2^ embryos at 12.5 dpc. At culture passage 4, they were plated and counted daily for 12 days to extrapolate growth curves. The mean values ± SE of three different cell clones (each originating from a different embryo) for each genotype are reported. **B.** Propidium iodide flow cytometry of asynchronous growing Ccdc6^wt/wt^ and Ccdc6^−ex2/−ex2^ MEFs. The percentage (expressed as mean value ± SE) of cells in each phase of the cell cycle is indicated. *P < 0.05. **C.** EMSA assay was performed with a radiolabelled oligonucleotide containing the CRE element incubated with nuclear extracts from Ccdc6^wt/wt^ and Ccdc6^−ex2/−ex2^ MEFs. **D.** Immunofluorescence of Ccdc6 in MEF wt, MEF^wt/−ex2^ and MEF^−ex2/−ex2^ was performed by utilizing the anti-Ccdc6 antibody (C-16-R).

To investigate the role of CCDC6 in cellular proliferation *in vivo*, MEFs from Ccdc6^wt/wt^ and Ccdc6^−ex2/−ex2^ embryos at 12.5 days post coitum (dpc) were obtained and the growth rate and cell-cycle distribution were analyzed. As shown in Figure 3A, the Ccdc6^−ex2/−ex2^ MEFs showed a proliferation rate significantly slower than the Ccdc6^wt/wt^ controls. To assess whether the slow rate of growth of Ccdc6^−ex2/−ex2^ MEFs was due to deranged progression through the phases of the cell cycle, we examined asynchronously growing MEFs by flow cytometry. Ccdc6^−ex2/−ex2^ MEFs accumulated in the G2 phase of the cell cycle, compared to Ccdc6^wt/wt^ MEFs cell cycle distribution (Figure 3B). Nevertheless, the percentage of the Ccdc6^−ex2/−ex2^ fibroblasts in the G1 and S phases was reduced with respect to MEFs Ccdc6 wild type.

Recent studies reported the involvement of the CCDC6 gene in apoptosis and the ability of its truncated mutant CCDC6 (1–101), which corresponds to the portion of CCDC6 included in RET/PTC1, to act as dominant negative on the wt CCDC6-induced apoptosis [7].

**Figure 4:**
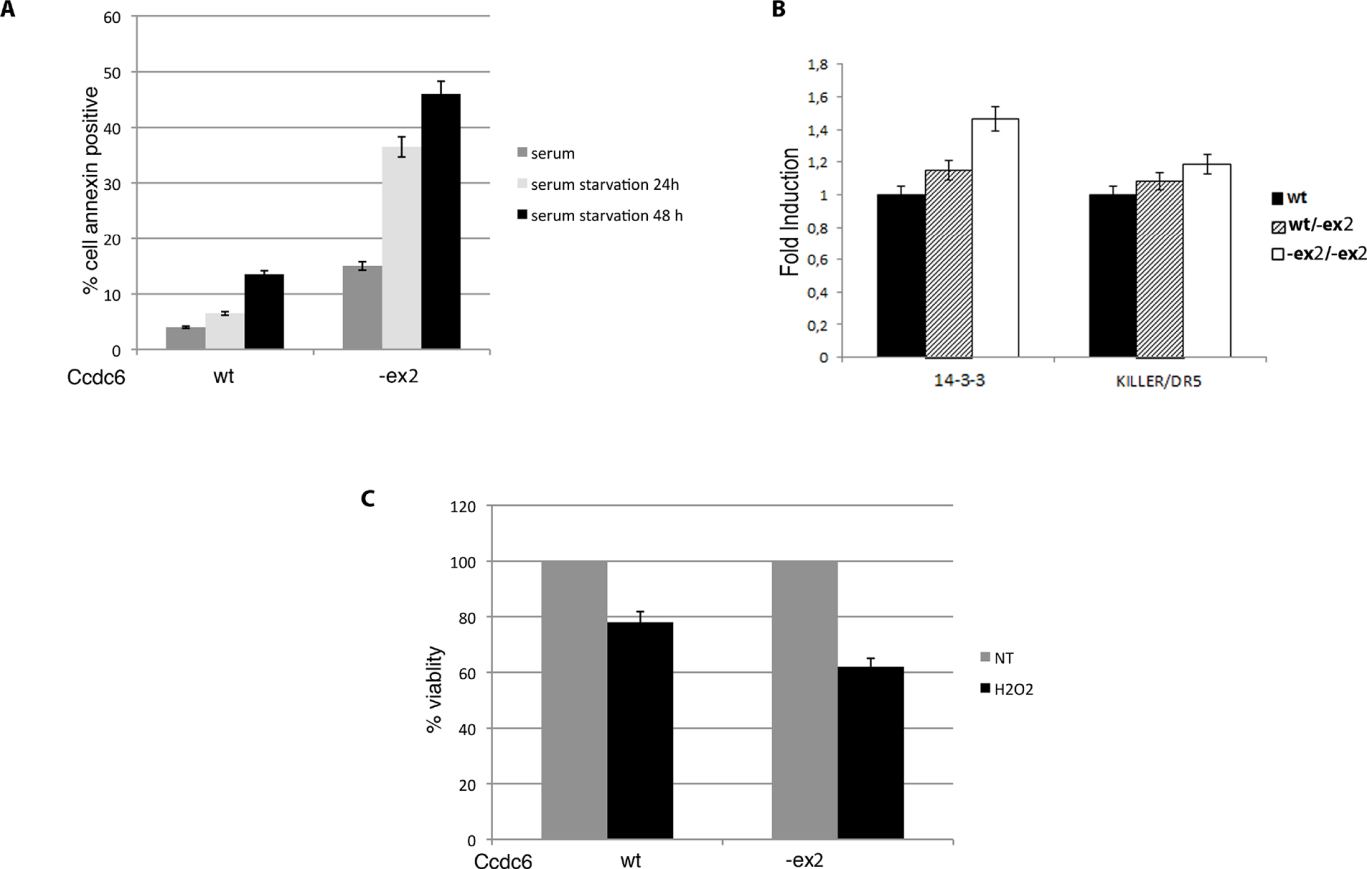
Ccdc6^−ex2/−ex2^ MEFs are more susceptible to apoptosis than wt MEFs **A.** Apoptosis analysis in Ccdc6^wt/wt^ and Ccdc6^−ex2/−ex2^ MEFs grown in complete medium or starved for 24 h or 48 h. Apoptosis was calculated as the percentage of cells positive at annexin V-FITC. **B.** 14–3-3σ and DR5 relative expression in MEFwt, MEF^wt/−ex2^ and in MEF^ex2/−ex2^ were analysed by qRT-PCR. Data are the mean ± SD of three independent experiments. **C.** Percentage of cell viability evaluated by WST-1 analysis on Ccdc6^wt/wt^ and Ccdc6^−ex2/−ex2^ MEFs treated or not with 800 mM H_2_O_2_. Values represent the mean of six experiments performed in duplicate.

Therefore, in order to evaluate the susceptibility of the Ccdc6^−ex2/−ex2^ MEFs to apoptosis we performed FACS analysis of MEFs derived from Ccdc6^wt/wt^ and Ccdc6^−ex2/−ex2^ embryos grown in complete medium or starved for 24 h or 48 h after annexin staining. As shown in Figure 4A (left panel) a consistent fraction of Ccdc6^−ex2/−ex2^ MEFs were annexin positive, 8-fold more than Ccdc6^wt/wt^ MEFs. Then, to confirm that Ccdc6^−ex2/−ex2^ MEFs had an increased susceptibility to apoptosis, we detected in unstressed Ccdc6^−ex2/−ex2^ MEFs, a significant increase in 14–3-3σ transcription, a regulator of apoptosis and cell cycle checkpoints, whose activity has been already linked to CCDC6, compared to wild type MEF [12]. Moreover, in Ccdc6^−ex2/−ex2^ MEFs, we also observed increased transcription of Killer/DR5, an effector of TRAIL, whose pathway is known to be active in thyroid, as already reported [13]. In Ccdc6^wt/−ex2^ MEFs the fold inductions of both the apoptotic proteins looked very similar to wild type MEFs (Figure 4B).

Next, we analyzed the susceptibility to the apoptosis of Ccdc6^wt/wt^ and Ccdc6^−ex2/−ex2^ MEFs by cell viability assay, evaluated by water-soluble tetrazolium salt (WST-1) analysis, after treatment with high doses of H_2_O_2_. As shown in Figure 4C the Ccdc6^−ex2/−ex2^ MEFs resulted sensitive to high doses of H2O2 (800 mM) showing a lower percentage (60%) of viable cells with respect to Ccdc6^wt/wt^ MEFs (78%) (Figure 4C). Nevertheless, it has been reported that MEFs show a high susceptibility to high doses of oxidative damage [14]. Finally, nuclear extracts from MEF Ccdc6^−ex2/−ex2^ showed an increased binding to CRE elements with respect to the extracts from the wt fibroblasts (Figure 3C). Interestingly, at immunofluorescence in MEFs Ccdc6^−ex2/−ex2^ the Ccdc6 protein showed a nuclear localization and a reinforced cytosolic staining, with respect to the wt and to the heterozygotes MEFs (Figure 3D).

### *Ccdc6-ex2 knock-in* mice develop thyroid hyperplasia associated with an increased CREB1 transcriptional activity

**Figure 5:**
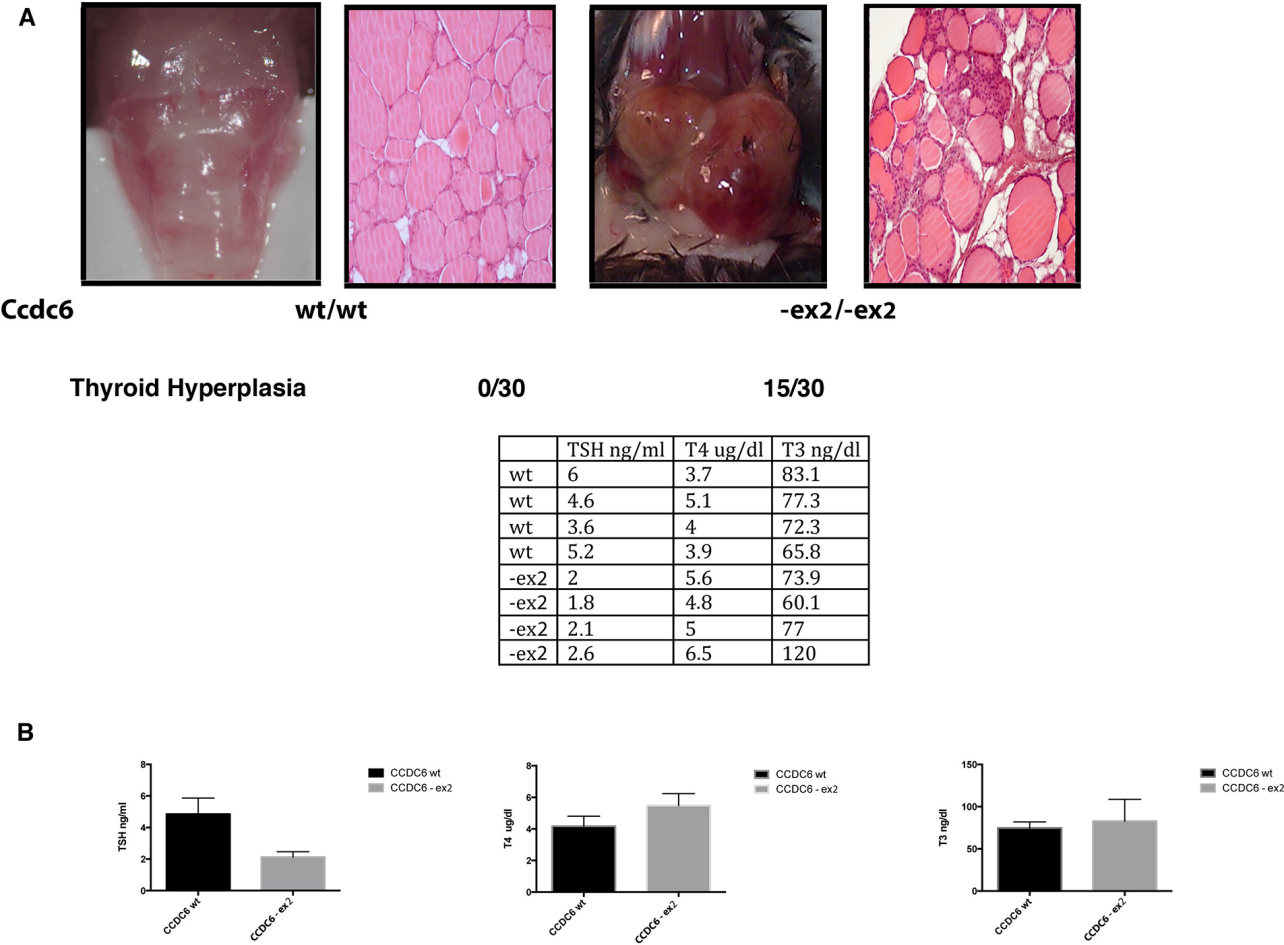
Ccdc6 knock-in mice develop thyroid hyperplasia **A.** Pictures show histology of a normal thyroid in Ccdc6 wt mouse with a single layer of cuboidal epithelium lines colloid-filled follicles and a hyperplastic thyroid in Ccdc6 knock-in mouse showing hyperplastic follicular cells arranged around amorphous, eosinophilic and inactive colloid. The Table 1 indicates the frequency of spontaneous hyperplasia in Ccdc6 knock-in mice. **B.** The table shows the values of TSH, T3 and T4 in Ccdc6 wt and Knock-in mice at 13 months of age. The histograms represent the mean of value ± SD.

Either Ccdc6^−ex2/−ex2^ or Ccdc6^wt/−ex2^ mice did not show any evidence of illness up to 12 months of age. However, histopathological analysis of aged (17–22 month-old) mice revealed the presence of thyroid hyperplasia in 15 out of 30 Ccdc6^−ex2/−ex2^ mice. Histologically, atypical follicles contained eosinophilic secretory and inactive material (colloid). Follicular cells were cuboidal to columnar with indistinct cell borders, moderate amount of eosinophilic cytoplasm, one basally located oval nucleus and indistinct nucleoli (Figure 5A). In Ccdc6^wt/wt^ mice the thyroids did not show any alteration, as well as in Ccdc6 ^wt/−ex2^ mice (data not shown). Consistently, we found low levels of TSH and high levels of T3 and T4 hormone levels in Ccdc6^−ex2/−ex2^ mice with respect to Ccdc6^wt/wt^ analyzed at the 13 month of age (Figure 5B).

**Figure 6:**
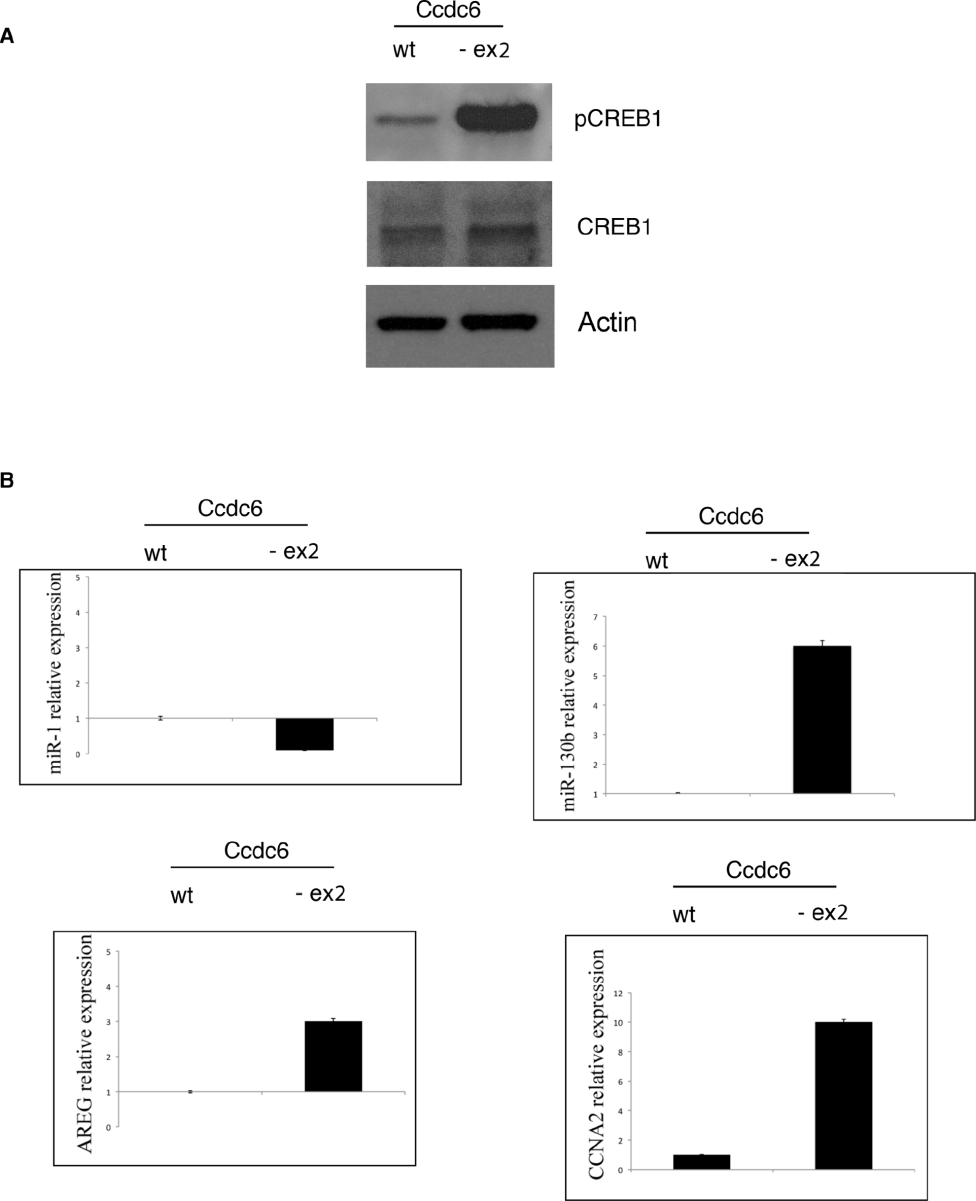
CREB1 transcriptional activity is increased in thyroids of Ccdc6^−ex2/−ex2^ mice **A.** Western blot analysis of pCREB protein in total cell extracts from Ccdc6^wt/wt^ and Ccdc6^−ex2/−ex2^ thyroids. Tubulin expression was used to normalize the amount of loaded proteins. **B.** AREG, CCNA2, miR-1 and miR-130b genes expression by qRT-PCR from Ccdc6^wt/wt^ and Ccdc6^−ex2/−ex2^ thyroids. Relative expression indicates the change in expression levels in comparison with wt tissue. Data are mean ± SD of three independent experiments.

We have previously demonstrated that CCDC6 was able to act as negative regulator of the transcriptional activity of CREB1 and of its phosphorylation [9]. As shown in Figure 6A, western blot showed increased levels of phosphorylation of CREB1 protein (at Ser 133) in extracts from hyperplastic thyroids obtained by Ccdc6^−ex2/−ex2^ mice with respect to the extracts from normal thyroid from wt mice. In order to investigate whether the increase in the phosphorylation status may reflect the transcriptional ability of the thyroid cells, we have analysed the levels of some CREB1 target genes, such as AREG, Cyclin A, miR-130b (positively regulated by CREB1) and miR-1 (negatively regulated by CREB1) in hyperplastic thyroids of Ccdc6^−ex2/−ex2^ mice and controls. As shown in Figure 6B, the CREB transcriptional targets AREG, Cyclin A and miR-130b showed an upregulated profile, whereas miR-1 appeared downregulated in Ccdc6^−ex2/−ex2^ mice with respect to the Ccdc6^wt/wt^ mice. These results confirm that the impairment of the Ccdc6 coiled-coil domains influences the CREB1 transcriptional activity in a positive fashion.

## DISCUSSION

Previous studies have demonstrated that the expression of CCDC6 is able to reduce CREB1 activity by increasing the PP1 activity on CRE element, with a consequent decrease of binding of CREB1 to the responsive regions and by activating HDAC1, affecting the acetylation status of histone H3 at the CREB1 responsive promoters [9]. Therefore, the negative modulation of the CREB1 activity, exerted by CCDC6, may account for the inhibition of cell growth as CREB1 activation represents the final step of the TSH activation pathway that exerts a critical role in the thyroid cell growth and differentiation [15, 16]. Thus, CCDC6 would work as tumor suppressor in inhibiting the CREB1-dependent gene expression. Therefore, the loss of one allele in the PTCs carrying the RET/PTC1 oncogene might amplify the RET/PTC1 oncogenic activity in leading thyroid cells to the malignant state, acting in synergism with the oncogene. The tumor suppressive role of CCDC6 in thyroid carcinogenesis is also supported by recent investigations showing that CCDC6 is involved in the protection of genome integrity. Indeed, silencing of CCDC6 or the mutation in the recognition site for the ATM kinase in primary GC1 germ cells increases the rate of survival, impairs the phosphorylation of histone H2AX on S139, affects ROS production and makes the cells more resistant to oxidative damage and genotoxic stress [17, 18].

In order to demonstrate a tumour suppressive role of the CCDC6 gene *in vivo* we first planned to generate a Ccdc6 null mice. Unfortunately, we could not generate mice null for Ccdc6 since we did not get germ-line transmission starting from the chimeric founder mice derived from embryonic stem cells harboring a targeted mutation in a single Ccdc6 allele (data not shown).

Next, we decided to generate Ccdc6 mice carrying a mutant Ccdc6 gene, altered in its function. To impair CCDC6 activity we deleted the exon 2 of the Ccdc6 gene that codes for almost the entire coiled-coil domain that has been demonstrated to be important for the activity of the fusion gene products that carry CCDC6 [1–5] and also for the transcriptional ability of the CCDC6 interacting partners [9]. By overexpressing a Ccdc6 mutant construct we verified that Ccdc6-ex2 protein was able to increase the CREB binding to the CRE element, but, at the same time, to exert a milder repression on CREB1 transcriptional activity with respect to wt Ccdc6 protein, suggesting that the deletion of the coiled-coil domain encoded by the exon 2 affects the CCDC6 ability to downregulate the CREB1 activity. Therefore, on the basis of this evidence, we generated a knock-in mouse in which Ccdc6-ex2 replaced the wt Ccdc6 gene. By performing the analysis of the mice phenotype we observed that the 50% of the Ccdc6^−ex2/−ex2^ mice (15/30) developed thyroid hyperplasia that was never observed in the wild type littermates (*N* = 30), in our study. Consistently, high levels of T3 and T4 and low levels of TSH were detected in the Ccdc6^−ex2/−ex2^ with respect to wt. Then, we demonstrated that the CREB1 transcriptional activity was increased in the thyroids of the Ccdc6^−ex2/−ex2^ mice. In fact, lysate extracts from hyperplastic thyroids of Ccdc6^−ex2/−ex2^ mice showed higher levels of CREB1 phosphorylation in Ccdc6^−ex2/−ex2^ mice in comparison with the wt counterpart at immunoblot with the anti-pS133 CREB1 antibody. Accordingly, in hyperplastic thyroids of Ccdc6^−ex2/−ex2^ mice, the expression analysis of CREB1 target genes revealed the upregulation of CREB1 associated to increased levels of AREG and Cyclin A, or decreased levels of miR-1, confirming the modulation of these gene by an enhanced CREB1 activity.

We have also analyzed other organs of the Ccdc6^−ex2/−ex2^ mice. In Ccdc6^−ex2/−ex2^ mice, out of the head and neck region, left ventricular hypertrophy has been rarely observed, even though without a statistical significance (data not shown). Moreover, a testicular phenotype has been observed occasionally in few animals deleted of exon 2 and a CCDC6 involvement in testicular germ cell population at normal and pathologic level has been reported [18]. Conversely, the Ccdc6^wt/−ex2^ mice analyzed did never show any pathology.

Thus, the animal studies confirmed that the loss of CCDC6 activity is able to increase the thyroid cell proliferation by amplifying the CREB1 activation and overall synergizing with the activity of the RET/PTC1 oncogene in the induction of the PTCs. The same mechanisms could be, of course, ascribed to the different oncoproteins that carries a truncated CCDC6 at their aminoterminus.

By the analysis of the MEF Ccdc6^−ex2/−ex2^ we detected a replication rate slower than the MEFs wild type. However, it has been well documented that to an enhanced CREB1 activity corresponded an increase of the growth rate in thyroid cells and a decrease in that of fibroblasts, due to apoptosis. Indeed, we observed an increase of the CREB activity that enhances the growth rate of thyroid cells, while decreases fibroblast proliferation [19–22]. Therefore, Ccdc6-ex2 leads to an increased CREB activity, but has a negative effect on MEFs proliferation. In conclusion, the results we report here are in support of a likely role of the haploinsufficiency of CCDC6 expression in the development of PTCs carrying the RET/PTC1 rearrangements.

## MATERIALS AND METHODS

### Expression vectors

The expression Ccdc6 wt (nt 1–1410) and Ccdc6-ex2 (Δnt 220–372) plasmids were obtained by amplifying the Ccdc6 full lenght or the ccdc6 deleted, in the nucleotides 220–372, by PCR and then inserting them into the *mcs* of the pcDNA4ToB (Invitrogen).

### Electrophoretic mobility shift assay

EMSA was performed as previously described [9]. CREB consensus oligonucleotide probe was from Santa Cruz Biotechnology Inc (TransCruz™ Gel Shift Oligonucleotides).

### Transactivation assay

B-CPAP cells were transiently transfected with the reporter construct [9]. Co-transfections were carried out in the presence of 200 ng of reporter construct and 500 ng of Renilla construct and 2 μg of pCMVSport6-CREB1 with or without the indicated amounts of wt Ccdc6 or mutant Ccdc6-ex2 constructs. Luciferase and Renilla activities were measured with the dual-luciferase reporter assay kit (Promega).

### Generation and genotyping of mutant mice

The mouse *Ccdc6* genomic locus (ENSMUSG00 000048701) was cloned screening a phage library of mouse 129/Sv genomic DNA (Stratagene, La Jolla, California) using a mouse *Ccdc6* cDNA as a probe, which was obtained by cross-hybridization experiments with human *CCDC6* cDNA. A targeting vector was constructed from subcloned genomic fragments. For the selection of targeted clones, a neomycin-resistance (*neo*) cassette was placed in the opposite transcriptional orientation to the endogenous *Ccdc6* gene. The final product consists of a 5′ genomic fragment (1.66 kb) containing exon 1, the *neo* cassette (1.7 kb) and a downstream *CCDC6* genomic fragment (5.73 kb). Thus, a ~12 kb *Ccdc6* genomic region containing exon 2 was replaced with the *neo* cassette vector.

Linearized targeting vector DNA was introduced by electroporation into 129/Sv embryonic stem (ES) cells. After G418 selection, genomic DNA of resistant clones was analyzed by Southern blot analysis using a probe lying within the 5′ homology region (generated with primers 5′-GCCCTTGTTGGTGGAGTGTT-3′ and 5′-ACCCTCTCTCCCTACCTACA-3′) hybridized to PstI digested genomic DNA. To generate chimeras, cells of targeted clones were injected into host blastocysts of C57BL/6J mice then transferred into uteri of pseudo-pregnant females. Male chimeras were mated with C57BL/6J females and F1 heterozygous progeny were intercrossed. Mice were genotyped for the *Ccdc6* deletion by PCR. Primers used for genotyping are as follows:

CCDC6 ln3R1 5′-GGAGGCAGATGAGTTCCTAAGG-3′

NEO5′R 5′-CTAAAGCGCATGCTCCAGACTGCC-3′

CCDC6U2 5′-CAGTAACACTTTATTCAAGAAAATCC AG-3′

### Growth and cell cycle analysis of MEFs

Primary MEFs were obtained from 12.5 day-old embryos. The MEFs were used to establish single cell suspensions. They were grown in Dulbecco's modified Eagle's medium (DMEM) (GIBCO) containing 10% fetal bovine serum (Hyclone), 1% glutamine (GIBCO), 1% penicillin/streptomycin and 1% gentamicin (GIBCO). The cells (4 × 10^5^/dish) were plated in a series of 6-cm culture dishes and counted daily with a hemocytometer for 12 consecutive days to extrapolate growth curves. Cell cycle analysis was performed using a FACS Calibur cytofluorimeter (Becton Dickinson, San Jose, CA, USA). For cell cycle analysis, MEFs in logarithmic growth were trypsinized, fixed in 70% ethanol and stored at 4°C for a few days. Cells were then washed with PBS without Ca^2+^ and Mg^2+^, stained with 50 μg/ml propidium iodide containing RNAse (20 μg/ml) and analyzed by FACS. Cell debris and fixation artefacts were gated out, and G1, S and G2/M populations were quantified using CellQuest software (Becton Dickinson). A similar number of events was analyzed in each experiment.

### Protein extraction, western blotting

Protein extraction and Western blotting procedures were carried out as reported elsewhere [23]. The indicated antibodies have been used for Western blotting: anti-CCDC6 antibody (C-16-R) has been generated upon rabbit immunization with the following polypeptide (Cys-Gly-Leu-His-Val-Gln-His-Met-Gly-Thr-Ser-His-Gly-Ile-Thr-Arg), synthetized by the NeoSystem Group, (Strasbourg, France) and affinity purified from immunized serum; anti-CREB1 has been obtained from Upstate Biotechnology; anti-pCREB Ser133 was from Cell Signaling. Anti-γ-tubulin (sc-17787) and anti-Actin (sc-1615) were from Santa Cruz Biotechnology. Blots were visualized using the ECL chemiluminescence system (Amersham/Pharmacia).

### RNA extraction, cDNA preparation, semiquantitative and quantitative reverse transcription-PCR

Total RNA isolation, RT-PCR and qRT-PCR from tissues or from cells were performed as previously described [24]. Primers used are as follows:

AREG(f) 5′-gcgaatgcagatacatcgag-3′;

AREG(r) 5′-ccacaccgttcaccaaagta-3′;

CCNA2(f) 5′-cttggctgcaccaacagtaa-3′;

CCNA2(r) 5′-caaactcagttctcccaaaaaca-3′;

14–3-3(f) 5′-tgtggacagccgacagtg-3′;

14–3-3(r) 5′-cttcagatgtgggggtcatc-3′;

Killer/DR5(f) 5′-ccctgagatctgccagtcat-3′;

Killer/DR5(r) 5′-tgggggtacaggaagtcagt-3′.

### Indirect immunofluorescence

The indirect immunofluorescence was performed as previously described [7].

### WST-1 colorimetric assays

To determine cell viability, a tetrazolium salt WST-1 assay was performed as directed by the manufacturer (Roche).

### Annexin assay

For Annexin V apoptosis assay (BD, Pharmingen, EremBodegem, Belgium), we stained cells simultaneously with fluorescein isothiocyanate (FITC)-Annexin V and non-vital dye PI according to the manufacturer's instructions. The percentage of the M-phase cells was determined by staining with PI and antibody to phospho-histone H3 (P-H3) (Cell Signaling, Beverly, MA, USA), followed by FITC-conjugated secondary antibody (Jackson Immunoresearch Laboratories, West Grove, PA, USA). Samples were analyzed with a CyAnTM ADP flow cytometer (Dako Cytomation, Glostrup, Denmark) using an argon-ion laser tuned to 488 nm measuring forward and orthogonal light scatter. Data were acquired by Summits software and analyzed with Modfits software.
